# An Exploratory Study of Quality of Life and Its Relationship with Academic Performance among Students in Medical and other Health Professions

**DOI:** 10.3390/medsci8020023

**Published:** 2020-06-09

**Authors:** Vijay Kumar Chattu, Pradeep Kumar Sahu, Neela Seedial, Gerlisa Seecharan, Amanda Seepersad, Melina Seunarine, Shivanna Sieunarine, Kahamaron Seymour, Samantha Simboo, Arissa Singh

**Affiliations:** 1 Department of Medicine, Faculty of Medicine, University of Toronto, Toronto, ON M5S 1A8, Canada; 2 Occupational Medicine Clinic, St. Michael’s Hospital, Toronto, ON M5C 2C5, Canada; 3 Institute of International Relations, The University of the West Indies, St. Augustine, Trinidad and Tobago; 4 Centre for Medical Sciences Education, Faculty of Medical Sciences, The University of the West Indies, St. Augustine, Trinidad and Tobago; pradeepmlg@gmail.com; 5 Department of Clinical Medical Sciences, Faculty of Medical Sciences, The University of the West Indies, St. Augustine, Trinidad and Tobago; neela.seedial@gmail.com (N.S.); gerlisa25@gmail.com (G.S.); amandaseepersad8@gmail.com (A.S.); melina.seunarine7@gmail.com (M.S.); shivanna_sieunarine@yahoo.com (S.S.); kahamaron.seymour@gmail.com (K.S.); simboo53@gmail.com (S.S.); arissasingh20@gmail.com (A.S.)

**Keywords:** quality of life (QOL), academic performance, grade point average (GPA), medical sciences, dentistry, veterinary medicine, optometry, pharmacy, ethnicity, WHO

## Abstract

Quality of life (QOL) is a broader concept which represents experiences, states, appraisals, behaviors, capacities and emotional reactions to circumstances. The study aimed to evaluate the differences in various domains of QOL among the students of five schools (medicine, dentistry, veterinary medicine, pharmacy and nursing) and an optometry unit in the Faculty of Medical Sciences, Trinidad and Tobago. Further, the study evaluated the factors (sociodemographic variables and academic performance) predictive of physical, psychological, social and environment domains of quality of life. The research tool consisted of a validated questionnaire which had two sections; (1) sociodemographics inclusive of students’ cumulative grade point average and (2) the shorter version of WHO quality of life (WHOQOL-BREF). The data were transformed into a linear scale and exported into the IBM SPSS version 24 where t-tests, one-way ANOVA and stepwise regression were performed. Of the total 535 participants, most 383 (71.6%) were females. While comparing the differences in the domains of QOL that existed based on the schools (professions) they were enrolled, significant differences were recorded for physical (*p* < 0.05), psychological (*p* < 0.05) social (*p* < 0.05) and environmental domains (*p* < 0.05). Though the domains of physical health, psychological health and environment showed a significant association with the academic performance of students, the social domain had no such relationship. The overall quality of life has a positive connection with the academic performance of students in medical and health professions. Therefore, universities and all stakeholders involved in health professions need to play a critical role to ensure the students in health professions maintain a high QOL. At the same time, there is a great need for extra attention for students who showed poor academic performance in the previous semester to bring them on track.

## 1. Introduction

Quality of life (QOL) is a leading concept that represents experiences, states, appraisals, behaviors, capacities and emotional reactions to circumstances [1]. WHO defines “quality of life as an individual’s perception of their position in life in the context of the culture and value systems in which they live and concerning their goals, expectations, standards and concerns” [2]. Currently, medical students in Trinidad and Tobago are experiencing increased levels of stress—including burnout and depressive symptoms [3]. Additionally, low QOL among these students is associated with unhealthy lifestyles, mental health problems and academic failure; thus, hurting professional development [4]. It is well-known that medical and health-profession careers are among the most challenging careers to pursue due to its high-intensity academic courses and training. Medical and health-profession students face an enormous amount of stress and burnout. These factors may disturb their QOL, therefore subsequently affecting their grade point average (GPA). High academic achievements greatly influence their professional competence in long term career, which accounts for them feeling pressured and having sensations of doing lengthy revision periods all the time. A study from Pakistan by Sarwar et al. reported that the mental health domain of QOL of medical students is suboptimal than physical health, especially among female students [5]. In a Brazilian study, medical students with depressive symptoms had lower scores in all domains of QOL and female students had lower health-related quality of life (HRQL) scores than males. There were significant impairments in HRQL observed among Year 3 students, students with depressive symptoms and among females [6]. It is often preached in medical school that students need a balanced life, which automatically results in high levels QOL. To do so, students must manage their time wisely and incorporate other activities along with studies, for example, exercise and enough sleep, which allows for the consolidation of memory and the desired GPA. A cross-sectional study from the Chinese Medical University using the Chinese equivalent of the WHOQOL-BREF to estimate the QOL of students found that the year of their study and the different specialties had notable differences in social and psychological domains (*p* < 0.05) which determined the QOL of the students [7]. The lowest scores were from the third-year students, while students from paraclinical years had the highest ratings in social and psychological domains. Third-year students are faced with a lot more academic stress comparable to students from other years of study as it was the year prior to clinical studies and therefore, showed a notable decline in QOL scores. A study from other regions also showed that some of the challenges faced by students include managing the psychosocial environment and financial problems, accompanied by academic pressures [8,9]. In this context, the current research project was designed to evaluate the differences in the domains of QOL based on students among the five schools, i.e., medicine, dentistry, veterinary medicine, pharmacy and nursing and an optometry unit in the Faculty of Medical Sciences, St. Augustine, Trinidad and Tobago. Further, the study aimed to evaluate the factors (inclusive of sociodemographic variables and academic performance) predictive of physical, psychological, social and environment domains of QOL.

The research questions of the study were as follows:

1. Are there any differences in the domains of QOL based on students studying in different schools/unit?
2. Are the domains of QOL as a dependent variable associated with the sociodemographic variables inclusive of GPA of students of Faculty of Medical Sciences, The University of the West Indies, St Augustine?

## 2. Materials and Methods

### 2.1. Study Design

The study was conducted among the students of the Faculty of Medical Sciences (FMS) at The University of the West Indies, St. Augustine, Trinidad and Tobago. A convenience sampling method (a type of non-probability sampling) was used where participants were selected because of their convenience, ease of accessibility and nearness to the researcher [10]. The inclusion criteria required that (1) the participants were willing to respond, and (2) were registered as a student of FMS. The study participants were met at their respective schools either before or after their lectures. The participation was voluntary, and the participants (students) were asked to complete the questionnaire after which the investigators would collect the completed forms. Alternatively, the questionnaires were given to the class representative who would distribute them to the class and was requested to receive the completed forms and return them to the investigators. The questionnaires were distributed over six (6) weeks during March and April 2019 before the end of the semester as most of the students were available that period. This study was approved by the Campus Research Ethics Committee (CEC) at the University of the West Indies, St. Augustine (Ref: CEC783/11/18).

### 2.2. Participants

The study sample composed of students currently enrolled in any of the five schools, (medicine, dentistry, veterinary medicine, pharmacy and nursing and optometry unit in the Faculty of Medical Sciences. A total of 535 participants completed the questionnaire of which, 152 were males (28.4%) and 383 were females (71.6%). Each participant’s questionnaire was assigned a number code to ensure the anonymity of participants and the confidentiality of the obtained information.

### 2.3. Instrument

The questionnaire consisted of two sections; (1) sociodemographics and (2) the shorter version of WHO quality of life (WHOQOL-BREF). The sociodemographic part included age, gender, marital status, school of study, nationality, ethnicity, family type and the number of siblings. It also included the respondents’ cumulative grade point average (CGPA), which was used to measure academic performance.

The second section consisted of the shorter version of WHOQOL-BREF, which comprises of 26 items and measures four domains: physical health, psychological, social relations and environment [11]. The tool follows a scoring system, where each response is rated on a 5-point Likert scale, ranging from option 1 (very dissatisfied/very poor/never) to option 5 (very satisfied/very good/always). These scores were transformed into a linear scale ranging from 0 and 100, with 0 being the least favorable and with 100 being the most favorable. WHOQOL-BREF was developed using data from the field-trial in 15 countries, and the instrument can be used in particular cultural settings, but at the same time, results are comparable across cultures. This instrument, by focusing on individuals’ own views and perceptions of their well-being status, provides a new perspective on the disease. The WHOQOL-BREF is a valid, reliable alternative to the assessment of domain profiles. In the current study, the Cronbach’s alpha coefficients in all four domains of QOL was higher than 0.70.

### 2.4. Data Analysis

The data collected was imputed into an excel sheet and then exported into the IBM SPSS Statistical software version 24. Frequency analysis was performed on the demographics to determine the number of participants and their variables such as age, gender, marital status, school, nationality, ethnicity, GPA, family type and the number of siblings. Student’s *t*-test was performed to find out any difference between male and female students in the domains of QOL. One-way ANOVA was performed to determine the differences in the domains of QOL based on students enrolled in different professional programs followed by the Bonferroni post hoc test. A p-value less than or equal to 0.05 was set as the threshold for statistical significance. The correlations between variables were calculated using Pearson correlation coefficients. A stepwise regression model was developed, with sociodemographic, students studying in different schools and GPA as independent variables and the QOL scores of four domains as dependent variables, to assess the adjusted relationship between the QOL and independent variables.

## 3. Results

### 3.1. Sociodemographic

A total of 535 students from the FMS responded to the questionnaire (Table 1). Most of the participants fell in the age of 18–21 years (52%) followed by 22–25 years (36.1%), 26–29 years (8.8%) and finally 30 and above (3.2%). Most students were single (81.9%), followed by other (14.6%) and married (3.5%). there were 170 MBBS (medicine) students, 98 dental students, 84 nursing students, 70 pharmacy students, 63 optometry students and 50 veterinary medicine students. the primary ethnic composition of the sample included East Indians (50.2%), Africans (22.1%) and mixed ethnicity (27.7%). A total of 492 students (92%) were from Trinidad and Tobago while the remaining 43 (8%) were non-nationals. In terms of GPA, the majority fell into the range of 3–3.59 (43.6%), followed by 3.6–3.99 (29.4%), 2.0–2.99 (18.9%) and lastly 4.0 and above (8.2%). In regards to the family type, it was found that the majority (63.6%) were having nuclear families and very few (12.1%) from extended families. It was found that most the students had two siblings (31.4%) and 3.6% of the students had no siblings.

### 3.2. Quality of Life (QOL) Based on Gender

Table 2 summarizes the comparative analysis of overall FMS students in the subdomains of QOL based on gender. Concerning, physical and psychological domains of QOL scores, no statistically significant differences between male and female students were recorded. However, the mean score for the social domain was significantly higher (*p* = 0.05) among male students (53.84 ± 23.47) compared to female students (49.16 ± 25.52). Similarly, a significant difference was found between male and female students for the environment domain as well (*p* = 0.03).

### 3.3. Quality of Life (QOL) and its Various Domains among Different Schools of Faculty of Medical Sciences

A one-way ANOVA was performed to determine if significant differences in the domains of QOL existed based on the students studying in different schools of FMS. Significant differences were recorded among the students in different schools concerning physical (*p* < 0.05), psychological (*p* < 0.05) social (*p* < 0.05) and environmental domains (*p* < 0.05). Comparison of the mean score of students from different schools of FMS in the domains of QOL is shown below (Table 3).

Bonferroni post hoc test for ANOVA shows a significant difference in the physical domain: MBBS and dental, dental and veterinary, dental and pharmacy, dental and nursing, dental and optometry (*p* < 0.05); psychological domain: MBBS and pharmacy, MBBS and nursing, MBBS and optometry, dental and optometry, veterinary and pharmacy, veterinary and nursing, veterinary and optometry, pharmacy and optometry, nursing and optometry (*p* < 0.05); social domain: veterinary and pharmacy; environmental domain: MBBS and dental, MBBS and veterinary, MBBS and nursing, MBBS and optometry (*p* < 0.05).

### 3.4. Correlations between Variables and Regression Analysis for Physical, Psychological, Social and Environmental Domains of QOL

Pearson correlation coefficients were calculated to find out the correlations between variables (Table 4). Further, in the stepwise regression analysis for physical, psychological, social and environmental domain of QOL (Table 5), the independent variables entered were: age, gender, marital status, school, nationality, ethnicity, GPA, family type and the number of siblings. The variables were shown to significantly predict the QOL of health profession students are reported in Table 5. Three independent variables, age (β = 0.881, *p* < 0.05), school (β = 0.396, *p* < 0.001) and GPA (β = 0.886, *p* < 0.05), were shown to be significant predictors of QOL—physical domain. For QOL—psychological domain, the regression analysis identified five independent variables, marital status (β = 1.059, *p* < 0.001), ethnicity (β = 1.102, *p* < 0.05), nationality (β = 2.836, *p* < 0.05), siblings (β = 0.704, *p* < 0.05), and GPA (β = 0.897, *p* < 0.05) were shown to be the significant predictors. For QOL social domain, marital status (β = 1.029, *p* < 0.001) and family type (β = 0.811, *p* < 0.05) were identified as significant predictors. In further regression, GPA (β = 0.862, *p* < 0.001), ethnicity (β = 1.012, *p* < 0.001), family type (β = 0.746, *p* < 0.05), age (β = 0.911, *p* < 0.05) and school (β = 0.411, *p* < 0.05) were shown to be significant predictors of QOL—environmental domain. Of the four regression models reported in Table 5, the regression of social domain reflected the weakest predictive value.

## 4. Discussion

The findings that medical students were more frequent victims of academic stress than non-medical students were expected, considering the more intensive study demands compounded by other background problems in a medical program compared to other programs. While the WHOQOL-BREF QOL scores for both males and females showed that the highest mean was seen in the environment domain, the social domain presented the lowest mean scores, which is contrasting to a Brazil study [12] which stated the opposite. It was observed that males showed higher mean scores compared to females in both social (53.84) and environmental (70.87) domains, respectively. However, studies from China [7], Saudi Arabia [13] and Pakistan [14] reported that males had higher scores than female students in the physical health and psychological health domains. This study also showed a statistically significant difference between males and females in the social (*p* = 0.05) domain of the WHO-QOL-BREF questionnaire. Males were found to have a markedly higher mean score than females in the domain of social relations. This is comparable to the Brazil study that also found males to have considerably greater mean scores in three out of the four domains evaluated, including the domain of the social relations [12]. A study was done from Saudi Arabia by Alkatheri et al., found that single students, males and smokers reported significantly lower QOL ratings in the social relationships domain [15]. In contrast, another study from Saudi Arabia concluded that the females in the medical program [16] are more stressed compared to males. Similar to a previous study done in Pakistan, our study indicated a positive relationship between QOL and being male, as they reported about the positive attitudes towards their interactions with friends and the support they provide to each other [13]. Additionally, statistically, a significant difference was found between males and females in the environment domain, and males were, again, found to have a higher mean score.

While comparing the differences in the domains of QOL existed based on the students studying in different schools of FMS, significant differences were recorded with respect to physical (*p* < 0.05), psychological (*p* < 0.05) social (*p* < 0.05) and environmental domains (*p* < 0.05). In the physical domain, Optometry students had the highest mean score (65.59) followed by veterinary medicine (63.57) and medicine (MBBS) (62.21) students. Whereas in psychological and social domains, Pharmacy students had the highest mean scores (72.62) and (60.83) followed by Nursing students (70.98) and (57.23). Further, concerning the environmental domain, MBBS students had the highest mean score, followed by the students from Pharmacy and other schools. Our findings are similar to those of several studies from India [17], Pakistan, [18] Thailand [19], Saudi Arabia [20] reporting examinations to be the most common source of academic stress for medical students. In a Sri Lankan study of 78 medical students, QOL scores were lower before the examination than scores after the examination. It is understandable that examination adds to mental and physical stress indicating students with better QOL had superior performance in examination as well [21]. Several studies have reported that psychological problems were common among medical students [22,23,24].

In the stepwise regression analysis for physical, psychological, social and environmental domain of QOL (Table 5), the independent variables entered were: age, gender, marital status, school, nationality, ethnicity, GPA, family type and the number of siblings. Concerning the physical domain, three independent variables, such as age, school and GPA, were shown to be significant predictors of the dependent variable. It indicates that academic performance, age and schools of students are significantly associated with the physical domain. Studies from Saudi Arabia [13] and the United States [25] shows a clear relationship between high achievers, scholastic accomplishments and physical environment such that high achievers were found to be more likely to inflict an affirmative remark on their physical activity. Thus, components of the environment domain such as a healthy physical environment, the opportunity for leisure activities and financial stability are positively correlated to academic excellence in the assessed Medical faculty.

For QOL—psychological domain, the regression analysis identified five independent variables, marital status, ethnicity, nationality and siblings were shown to be the significant predictors. Additionally, the academic performance of students is significantly associated with the psychological domain. Few other studies [26,27] have also reported inflation in GPA was attributed to a rise in the level of spirituality, intellect, drive and self-esteem and a drop in depression. High psychological health and good academic performance are positively linked to academic performance as it is associated with an increase in the GPA and associated with fulfilling highly demanding components essential for the success of future doctors [13]. Studies from Saudi Arabia concluded that the females in the dental program [28,29] are more stressed compared to males as these psychological disturbances negatively correlated with the QOL, leading to lower academic performance. To support this argument, another study from China study [8], found the nursing Student’s to have the least amount of scores in the psychological health domain and suggested likely causes such as anxiety and stress due to the poor relationship between doctor and patient as well as unemployment in China.

For QOL—social domain, marital status and family type were identified as significant at the *p* < 0.05 level. In further regression, ethnicity, family type, age and school were shown to be significant predictors of QOL—environmental domain. Research from Pakistan [14] compared medical and dental students and found the latter to have better results in the social relationship domain of QOL. Further, academic performance is also significantly associated with the environmental domain. The Saudi study [13] reported no significant difference between males and females concerning the environment domain. In contrast, a study from Pakistan found female dentistry students had a better quality of life than male students and were statistically significant in the environment domain [14]. Of the four regression models reported in Table 5, the regression of social domain reflected the weakest predictive value. The low GPA mean score can be attributed to stressors as documented by Naidu et al. in Trinidad which showed that “fear of failing course or year” and “examinations and fear of failing” were the highest-ranked stressors for dentistry students [26]. A study from St. Lucia [30] in the Caribbean region on stress and coping strategies among undergraduate medical and nursing students found that the commonly used coping strategies were active coping, positive reframing, planning and acceptance. Since medical education is very stressful, which could affect their health and well-being, there is a great need to study the stress and coping strategies among the medical and other health sciences. This assessment helps to take appropriate measures such as counseling/academic advising at a personal level to address the curriculum, assignments, course loads, provision of various learning platforms at a broader scale. The role of academic/student advisors is very critical for giving proper and timely advice to the students so that they can have a balance of their studies and life by developing right coping strategies.

## 5. Conclusions

In summary, the WHOQOL-BREF proved to be an excellent and reliable tool in this study of QOL. The domains of physical, psychological health and environment showed a significant correlation with the academic performances of students while social domain did not show any in our study. The overall QOL has a relationship with the academic performance of health professions students and thus educational institutes, universities, ministries of higher education and all stakeholders involved in medical and health professions education has a pivotal role in maintaining high QOL of students in health professions.

## Figures and Tables

**Table 1:** Sociodemographic characteristics of the participants.

Participants	Characteristics	N	%
Age	18–21	278	52
	22–25	193	36.1
	26–29	47	8.8
	30 and above	17	3.2
Gender	Male	152	28.4
	Female	383	71.6
Marital status	Single	438	81.9
	Married	19	3.5
	Other	78	14.6
School	MBBS	170	31.8
	DDS	98	18.3
	DVM	50	9.3
	Pharmacy	70	13.1
	Nursing	84	15.7
	Optometry	63	11.8
Nationality	Trinbagonian	492	92
	Non-national	43	08
Ethnicity	East Indian	269	50.2
	African	118	22.1
	Mixed and others	148	27.7
GPA	4 and above	44	8.2
	3.6–3.99	157	29.4
	3–3.59	233	43.6
	2.0–2.99	101	18.9
Family type	Nuclear	340	63.6
	Extended	65	12.1
	Single parent	103	19.3
	Others	27	5
Siblings	None	19	3.6
	01	69	12.9
	02	168	31.4
	03	140	26.2
	4 and above	139	25.9
Total		535	100

**Table 2:** Comparative analysis for the domains of the WHO quality of life (WHOQOL-BREF) among males and females.

Gender	Physical	Psychological	Social	Environment
	Mean ± SD	Mean ± SD	Mean ± SD	Mean ± SD
Male	60.57 ± 17.27	65.78 ± 20.15	53.84 ± 23.47	70.87 ± 19.98
Female	58.67 ± 15.69	64.20 ± 17.50	49.16 ± 25.52	66.69 ± 19.93
*p*-value	0.22	0.37	0.05 **	0.03 **

**—significant at 0.05 level of significance.

**Table 3:** Comparative analysis of faculty of medical sciences students in the domains of QOL.

School/Unit		Physical	Psychological	Social	Environmental
MBBS	M ± SD	62.21 ± 14.97	62.52 ± 17.77	55.64 ± 16.58	75.46 ± 18.26
Dental	M ± SD	46.83 ± 10.40	67.60 ± 14.71	55.95 ± 17.09	67.98 ± 13.85
Veterinary	M ± SD	63.57 ± 16.44	59.67 ± 19.22	49.50 ± 17.20	66.13 ± 18.99
Pharmacy	M ± SD	57.96 ± 14.44	72.62 ± 14.82	60.83 ± 18.83	73.48 ± 15.72
Nursing	M ± SD	60.80 ± 15.94	70.98 ± 17.15	57.13 ± 20.95	67.82 ± 15.93
Optometry	M ± SD	65.59 ± 18.27	50.07 ± 19.75	54.89 ± 18.85	66.42 ± 15.72
	F Value	18.30	16.27	2.43	5.81
	*p*-value	0.000 *	0.000 *	0.034 *	0.000 *

*—significant at *p* = 0.05 level.

**Table 4:** Pearson correlation for variables included in regression analysis.

Variable	1	2	3	4	5	6	7	8	9	10	11	12	13
1	1.00												
2	0.02	1.00											
3	0.05	0.30 **	1.00										
4	0.02	−0.02	0.21 **	1.00									
5	0.15 **	−0.03	−0.04	−0.05	1.00								
6	−0.03	0.04	0.11 *	−0.01	−0.06	1.00							
7	0.00	0.01	−0.01	0.31 **	−0.05	−0.06	1.00						
8	0.03	−0.18 **	−0.04	0.09	0.07	−0.12 **	0.02	1.00					
9	0.11 *	−0.15 **	−0.25 **	−0.01	0.02	−0.04	0.04	0.17 **	1.00				
10	−0.12 **	0.05	−0.02	0.10 **	−0.09 *	−0.07	−0.10 *	−0.06	0.03	1.00			
11	0.01	0.04	0.18 **	−0.04	0.11 *	−0.10 *	−0.09 *	0.01	0.05	0.38 **	1.00		
12	−0.05	0.05	0.14 **	0.03	0.02	−0.01	0.01	−0.11 *	0.02	0.28 **	0.43 **	1.00	
13	−0.10 *	0.06	0.04	−0.15 **	0.01	−0.13 **	−0.18 **	−0.11 *	−0.08	0.51 **	0.48 **	0.30 **	1.00

1—age, 2—gender, 3—marital status, 4—school of study, 5—nationality, 6—ethnicity, 7—GP, 8—family type, 9—siblings, 10—physical domain, 11—psychological domain, 12—social domain, 12 environmental domain. **—significant at 0.01 level, *—significant at 0.05 level.

**Table 5:** Summary of stepwise regression analysis for variables predicting physical, psychological, social and environmental domains (N = 535).

Dependent Variable	Independent Variable	Coefficient	Standard Error	*t*	*p* Value	95% Confidence Interval	
		β				Lower Bound	Upper Bound
QOL-Physical domain	Age	0.881	−0.127	2.998	0.003 *	−4.372	−0.991
	School	0.396	0.147	−3.297	0.001 **	0.527	2.083
	GPA	0.833	0.141	3.161	0.002 *	0.996	4.269
QOL-Psychological domain	Marital Status	1.059	0.216	4.974	0.000 **	3.187	7.348
	Ethnicity	1.102	−0.119	2.824	0.005 *	−5.279	−0.948
	Nationality	2.836	0.103	2.454	0.014 *	1.389	12.532
	Siblings	0.704	0.103	2.390	0.017 *	0.299	3.065
	GPA	0.897	−0.090	−2.134	0.033 *	−3.675	−0.152
QOL-Social domain	Marital Status	1.029	0.140	3.280	0.001 **	1.354	5.396
	Family Type	0.811	−0.109	−2.564	0.011 *	−3.670	−0.486
QOL-Environmental domain	GPA	0.862	0.164	3.752	0.000 **	1.541	4.930
	Ethnicity	1.012	−0.153	−3.656	0.000 **	−5.689	−1.712
	Family Type	0.746	−0.122	−2.912	0.004 *	−3.638	−0.707
	Age	0.911	−0.103	−2.469	0.014 *	−4.040	−0.459
	School	0.411	−0.087	−1.997	0.046 *	−1.627	−0.013

**—significant at 0.001 level, *—significant at 0.05 level.
